# Sharing goals by timely communication improves fishermen's satisfaction with marine protected areas: A case study in the Mediterranean

**DOI:** 10.1007/s13280-021-01683-y

**Published:** 2022-01-06

**Authors:** José Manuel Perea-Muñoz, Austin Miles, Just Tomàs Bayle-Sempere

**Affiliations:** 1 Departamento de Producción Animal, Universidad de Córdoba, Campus Rabanales, 14711 Córdoba, Spain; 2 Charles River Conservancy, 43 Thorndike Street, S3-3, Cambridge, MA 02141 USA; 3 Departamento de Ciencias del Mar y Biología Aplicada - Instituto Multidisciplinar de Estudio del Medio “Ramón Margalef”, Universidad de Alicante, POB 99, 03080 Alicante, Spain

**Keywords:** Effective communication, Fisheries, Marine conservation, Marine protected areas, Relational coordination framework, Supportive relationships

## Abstract

Marine protected areas (MPAs) are considered as a valid tool for mitigating the impact of fishing on marine ecosystems. Their success depends upon their acceptance by implicated stakeholders and on the integration of the stakeholder groups into their management. This integration is especially important with regard to fishermen, whose interests are the most directly affected by MPAs. The relational coordination method posits that effective communication and supportive relationships among stakeholders result in positive stakeholder behaviors and a more effective management of the system. Applying its principles, we designed a survey to evaluate the satisfaction of fishermen associated with five MPAs in the Spanish Mediterranean and determine what mechanisms affect fishermen’s acceptance of MPAs. Our results demonstrate that effective communication is particularly important for good supportive relationships and satisfaction among fishermen and other stakeholder groups, as well as satisfaction with the MPA. Sharing objectives with fishermen through timely communication is the primary mechanism to improve fishermen's satisfaction and ameliorate perceptions towards MPA. To address this issue, we recommend more substantial integration of fishermen in the co-management of MPAs.

## Introduction

Marine protected areas (MPAs) have become an important component of efforts to address overfishing and habitat degradation in the Mediterranean (Coll et al. 2010). They are also considered as a solution to the perceived failure of typical management methods (Kaiser 2005). MPAs achieve their goals by regulating permitted uses in such a way that balances stakeholder’s economic interests with habitat and species conservation (Di Lorenzo et al. 2020). Both fishery conservation and enhancement are principal objectives of MPAs and as such fishermen are one of the principal stakeholders involved in MPA management (Smallhorn-West et al. 2020). An important aspect of MPAs is how effective (in the ecological, economic, and social sense) is their management, considering that MPAs are often implemented in contexts that involve conflicting objectives (e.g., ecosystem conservation vs. fisheries enhancement; Carr 2000). The effectiveness of an MPA depends in large part on the support it receives from the local community, and that support is mainly obtained through inclusion of different actors in the process of design and management, which are expected to generate stakeholder satisfaction (Lundquist and Granek 2005) in contexts that are characterized by the conditions outlined by Gittell (2006):

1. *Task interdependence—*Many tasks depend on each other for success. For instance, compliance with rules and regulations is necessary for the MPA to achieve its ecological objectives, and their application is the responsibility of the managers. However, the participation of stakeholders can contribute to compliance because it will build awareness about the benefits of the MPA. Both compliance and participation altogether, among others management mechanisms, amplify synergistically the effectiveness of MPA management (Giakoumi et al. 2018).
2. *Uncertainty*—Ecosystems are complex entities about which much remains unknown. The assumptions underlying adaptive management, for instance ground it in the admission that humans do not know enough about ecosystems to manage them (Lee 2001).
3. *Time constraints*—Both internal (e.g., the managers need to demonstrate the benefits of MPAs in a determined period of time) and external (e.g., many international agreements have set deadlines for conservation goals, including for marine conservation, therefore there is the obligation to conserve a given portion of all marine resources before a given date).

Satisfaction is a critical factor for the performance of any process or system (Mirvis and Lawler 1977), therefore, it is important to investigate how MPA management can cultivate high levels of satisfaction among implicated stakeholders. Fishermen are particularly important and their involvement in different stages of design and management of MPAs has received particular emphasis in the last 30 years (Christie et al. 2017). Studies on the assessment of the attitude of different groups of stakeholders of MPAs in the Mediterranean are scarce because, unlike ecological benefits, the evaluation of socioeconomic impacts is more complex owing to the difficulty of gathering information about costs and revenues (Sanchirico et al. 2002), and to the heterogeneity of the effect of social impacts on different stakeholder groups and among individuals within each group (Mascia et al. 2010; McNeill et al. 2018). Some results indicate that different stakeholders do not necessarily share the same perspectives regarding MPAs, particularly in places in which various socioeconomic activities compete with each other (e.g., fishing and tourism; Pascual Fernández 2004) or in which individuals either within or among stakeholder groups come from different socioeconomic and demographic contexts (McClanahan et al. 2021).

Different studies have demonstrated that following the guidelines of relational coordination framework (Gittell 2006) increases satisfaction which in turn facilitates the achievement of the desired objectives in systems characterized by high levels of task interdependence, uncertainty, and time limitations, like MPAs. According to the relational coordination framework, productive cooperation is based on effective communication and supportive relationships which tend to promote positive behaviors among the participants in a system (Gittell 2006). Effective communication refers here to its frequency, timeliness, accuracy, and the degree to which it focuses on the resolution of problems rather than on placing blame. Supportive relationships, on the other hand, are defined by the degree to which they involve shared objectives, shared knowledge, and mutual respect (Gittell 2006).

A previous study (Miles et al. 2020) evidenced the low levels of satisfaction among key stakeholder groups (fishermen, divers, scientists, and managers) implicated in the functioning of the MPAs in the Spanish Mediterranean included in this study, and a low level of cooperation among those stakeholders. Significant fragmentation among different stakeholder groups was observed, particularly between those with economic interests in the MPA (fishermen and divers), and the management groups, (managers and scientists). Specifically, fishermen have the least favorable perceptions with regard to divers (Miles et al. 2020) because they compete directly for access to the MPA (Lucrezi et al. 2017) and generate some conflicts in the management of the MPA (Hogg et al. 2019). Communication channels were non-existent, the four considered stakeholder groups had poor relationships both within and among groups, and management structures did not give rise to general acceptance of the MPA from fishermen, SCUBA dive centers, and scientists.

Satisfaction influences the attitude of stakeholders towards MPAs which significantly will affect management outcomes (Giakoumi et al. 2018). Nonetheless, we do not know enough about the extent to which satisfaction is affected by the lack of cooperation among stakeholder groups to determine what actions would merit the effort to improve effective communication and supportive relationships among all these stakeholders; nor do we know which mechanisms of cooperation produce higher or lower levels of satisfaction and therefore cannot identify where efforts should be directed to improve the management and performance of MPAs via the increase of stakeholder satisfaction. As anticipated above, the stakeholders that put the most pressure on MPAs are fishermen, owing to the stringent regulation of extractive activities like fishing (Zupan et al. 2018) and the economic implications of restricting natural resource extraction (Badalamenti et al. 2000). Therefore, their attitude would have the most significant impact on the success of MPA management (Cicin-Sain and Belfiore 2005). An analysis of the system composed by the MPAs using the relational coordination framework would identify those specific mechanisms that drive effective communication and supportive relationships. Identifying those mechanisms in turn would yield recommended actions that would improve satisfaction with the MPA and will subsequently improve success.

With these goals in mind, the aims of this study are to (1) evaluate the contribution of cooperation boosted through effective communication and supportive relationships to the satisfaction of fishermen with the MPAs and (2) determine the specific mechanisms of cooperation that would improve the satisfaction of fishermen with MPAs and with other stakeholder groups. To these ends, we developed a model of structural equations based on the links between indicators of effective communication and supportive relationships with indicators of satisfaction of the fishermen with MPAs, on one hand; and with the other stakeholder groups on the other hand. Structural equation modeling (SEM; Boslaugh et al. 2008) is an ideal exploratory methodological framework for this type of study because it simultaneously allows for the emergence of latent factors and the modeling of links among those factors. Additionally, it can simultaneously identify the mechanisms that affect satisfaction and quantify their effect. Our results will be helpful as a framework for the design of future studies aiming to understand stakeholder satisfaction with MPAs or other environmental conservation contexts. They would be applicable too for the improvement of MPA planning and management because they suggest which mechanisms among actors should be using the relational coordination framework to enforce the MPA management effectively.

## Materials and methods

### Locations and study design

**Fig. 1:**
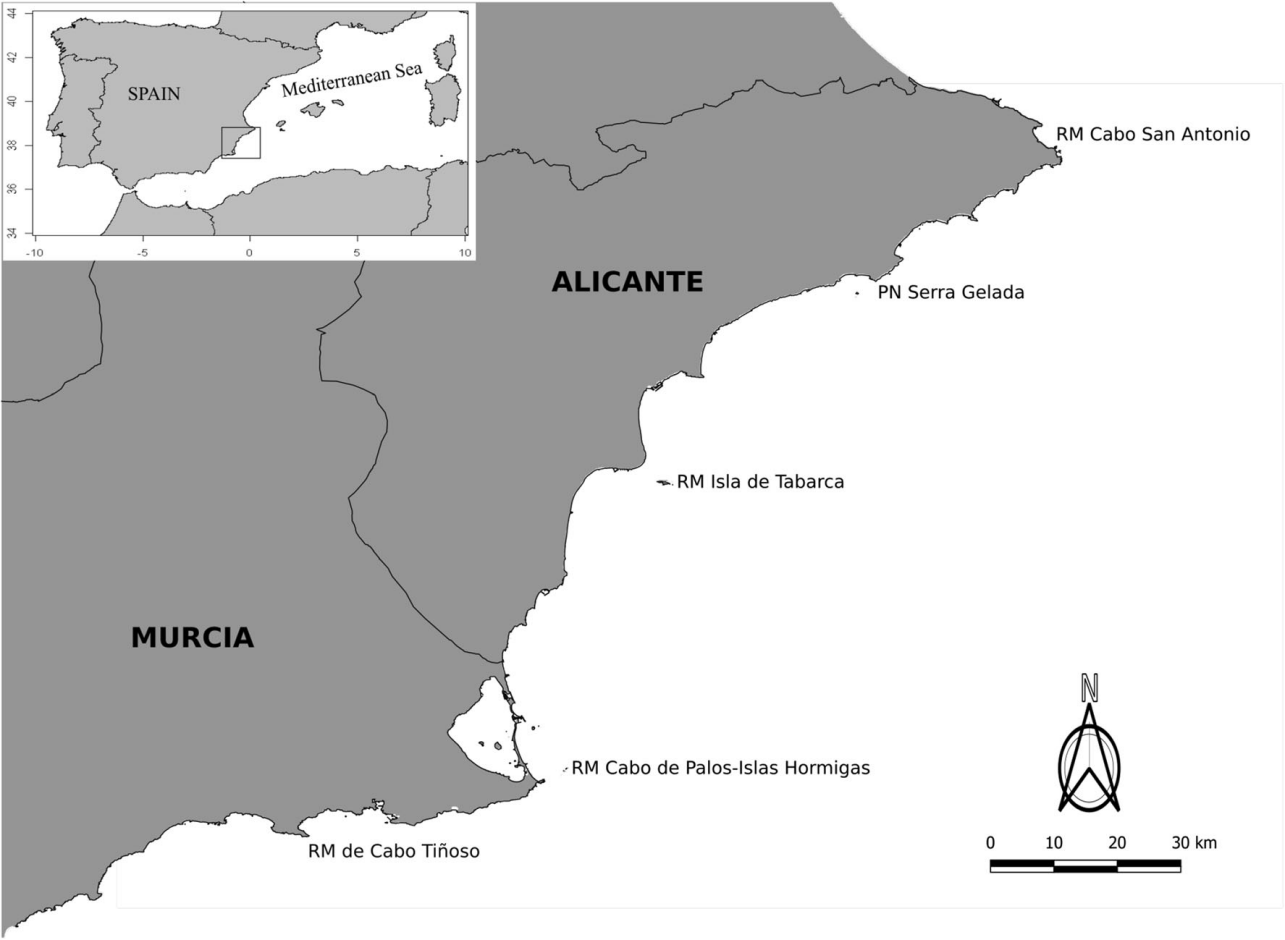
Map of the MPAs involved in this study

The study involved surveys delivered to fishermen associated with 5 of the 19 MPAs in the Spanish Mediterranean: Serra Gelada, San Antonio, Tabarca, Cabo de Palos, and Cabo Tiñoso (Fig. 1). These MPAs were chosen because they are located in the same biogeographical zone, are used on a regular basis by professional fishermen, and are designed according to the same criteria of zoning, regulations on use, and mechanisms of participation. We also consulted with the Administrations that manage the MPAs to assess the level of communication between managers and the other stakeholder groups. They explained that a management committee, which is a participatory body where the actions to be taken are communicated and discussed, convenes once a year. However, the fisheries administration makes the final decision, rendering the management committee effectively powerless (Hogg et al. 2017). Additionally, there is no more systematic and regular communication with any of the stakeholder groups involved in the MPAs to transmit guidelines, collect opinions, or agree on day-to-day management decisions. Accordingly, we consider that all the MPAs analyzed have a similar level of communication between managers and the rest of the stakeholder groups. Choosing MPAs on this basis ensures that the fishermen’s perception of each MPA would not be affected by variation in these factors among different MPAs.

Four primary stakeholder groups are associated with the marine protected areas: fishermen, SCUBA dive centers, scientists, and managers. Fishermen and dive centers are “user groups” that practice consumptive activities in or around the MPAs, and therefore stand to benefit economically from them. The activity of fishermen and dive centers is regulated by the managers, while the scientists carry out the ecological monitoring of the MPA. The fishing fleets studied vary substantially among different ports, ranging from the two vessels in Benidorm to the 36 in Santa Pola. In the ports where there are fewer than ten vessels, all fishermen were surveyed. In the ports with more than ten vessels, a representative sample of fishermen were surveyed, which would range from 60 to 80% of the total number of vessels included in the census of the fishermen’s Cofradías.

Data were gathered through surveys delivered in person to fishermen. The survey comprised 35 questions that used a 5-point Likert scale, in which the higher scores indicate more agreement with the statement given. At the end of the survey, respondents had time to add any other comments they felt were relevant. The relational coordination framework considers effective communication and supportive relationships as the two main components which indicate cooperation among individuals (within and among stakeholder groups) and determine the level of satisfaction. They were measured by means of seven dimensions developed by Gittell (2006): timely, accurate, frequent, and problem-solving talks characterize effective communication, while shared knowledge, shared goals, and mutual respect characterize supportive relationships. Fishermen were asked to answer questions regarding the behavior and perceived beliefs of all stakeholder groups. The satisfaction of the fishermen was measured using seven questions—three regarding the satisfaction with the MPA (with regard to its objectives, its current socio-ecological state, and its management), and four regarding the satisfaction with the four stakeholder groups (other fishermen, SCUBA diver centers, scientists, and managers). Surveys were delivered to 60 fishermen, who were listed in the censuses of vessels provided by the fishermen’s Cofradías at the ports close to the MPAs included in this study. All professional fishing vessels were included in an official registry (https://www.mapa.gob.es/es/pesca/temas/registro-flota/) and there are no vessels that carry out fishing on an irregular basis. These numbers represent 68.9% of the fishermen implicated regularly in the MPAs analyzed in this study which constitutes a relatively high proportion of the target population. The fishermen surveyed fall within a wide age range (between 29 and 60 years old), report different levels of benefit yielded from MPAs, and spend varying amounts of time and effort fishing close to the MPA.

### Statistical analysis

**Fig. 2:**
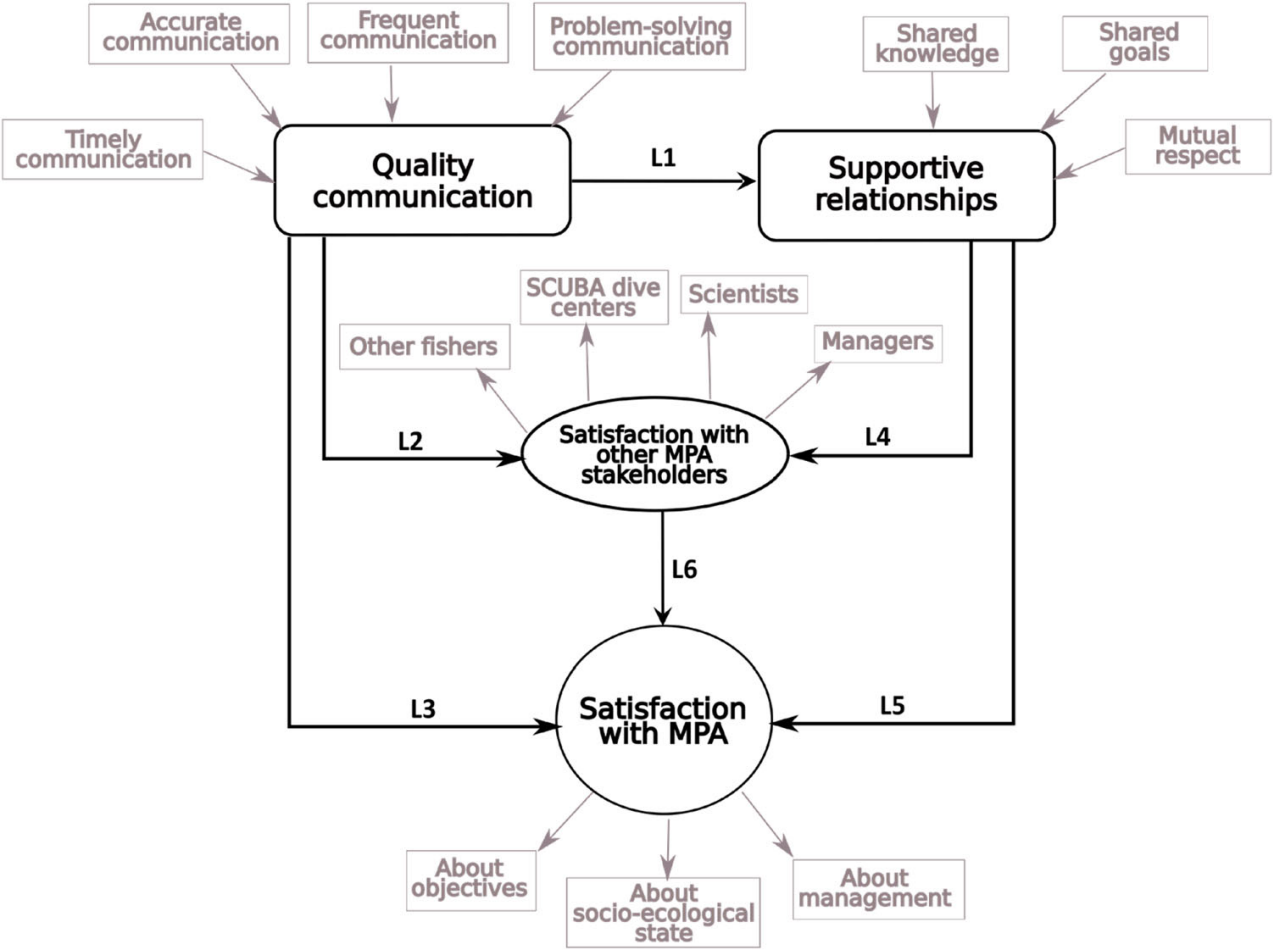
Proposed theoretical model and hypotheses

Based on a literature review (Gittell 2006; Miles et al. 2020), we developed a theoretical exploratory model applying relational coordination framework to assess the satisfaction with the MPA, as shown in Fig. 2. Following the assumptions of the relational coordination framework, the theoretical exploratory model proposes that a dynamic of effective communication and supportive relationships with fishermen reinforces satisfaction with other stakeholder groups and with the MPA. This increase of satisfaction would be the basis to improve the efficiency of the management of the MPA (Giakoumi et al. 2018).

Structural equation modeling (SEM) examines a theoretical exploratory model (Meydan and Sesen 2015). The model identifies potential multivariate links within and between the observable and latent variables from an inductive scope. The SEM method allows the examination of a complete model by generating goodness-of-fit statistics and evaluating the overall fit. The proposed links can be rejected as reasonable approximations of reality but cannot be confirmed as the exclusive representation of the actual underlying processes.

The model parameters were estimated using the robust unweighted least squares method (RULS-SEM; a variant of PLS-SEM) using LISREL 9.30 software. We opted to use RULS-SEM because the estimations are more robust with small sample sizes and Likert scales of measurement. The parameters were estimated from the asymptotic covariance matrices and polychoric correlations for ordinal indicators and we conducted oversampling using a bootstrapping technique to ensure the stability of results. Given the limited number of observations (60 fishermen) for the large number of variables, 100 bootstrap sub-samples were generated to validate the estimated model.

Initially, the model postulated in Fig. 2 was specified in the analytical procedure and its capabilities were evaluated to predict the target constructs. Several recommended goodness-of-fit indices were used to evaluate the fit of the model, including the Satorra–Bentler scaled *χ*^2^ likelihood ratio (Satorra and Bentler 1994), the root mean square error of approximation (RMSEA = mean-squared difference between observed covariances and model implications), the comparative fit index (CFI), and the normalized fid index (NFI). An RMSEA value of less than 0.08 suggests a satisfactory fit, and a value of less than 0.05 suggests a certain fit (Browne and Cudeck 1993). The NFI and CFI indices range from 0.0 to 1.0, in which higher values indicate better fit. As a point of reference for a good fit, a value of 0.90 is typically used (Kline 2005). In addition to the general goodness-of-fit measures, the null hypotheses of the individual parameters of the SEM that are different from zero were tested through the inspection of the Wald statistic (Kline 2005). The estimations of the parameters were obtained as if the variances of the variables were unitary. The multicollinearity of the models was verified using the variance inflation factor (VIF). A serious problem related to collinearity is considered to arise when the FIV values are higher than 10. After checking the fit of the estimated model, it was modified until the most plausible model was found (that with the highest level of agreement between the data estimated by the model with the observed data).

The goodness of fit for the measurement model was assessed using two statistical techniques. The Reliability Cronbach’s Coefficient of Alpha was used to calculate the reliability value of the three parts of the questionnaire, and the consistency of latent variables. We found that the three parts of the questionnaire—communication, relationships, and satisfaction—had reliability values of 0.85, 0.79, and 0.75, respectively. The average extracted variance was used to calculate the convergent validity of the latent variables. All these results will contribute to achieve Objective 1.

Following the proposed theoretical exploratory model, we suggest three blocks of potential multivariate links between latent variables (see Fig. 2). They relate to effective communication among fishermen and the other stakeholder groups which includes its consequences on supportive relationships among the four stakeholder groups (L1), the satisfaction of fishermen with the other three stakeholder groups (L2), and the satisfaction of fishermen with the MPAs (L3). The second block involves the influence of supportive relationships among all the stakeholder groups on the satisfaction of fishermen with the other stakeholder groups (L4) and the satisfaction of fishermen with the MPAs generated by supportive relationships (L5). The third block concerns the satisfaction of fishermen with the MPAs derived from the satisfaction with the other stakeholder groups (L6). The findings of this part will fulfill objective 2.

### Limitations

A relatively small number of collected surveys could limit the scope of this study. It is always desirable to have a large sample size; however, in our study, it would be impossible to obtain a much larger sample size because there are only 87 fishermen associated with the MPAs included in this study, of which we surveyed 60 (68.9%).

For some authors, the sample sizes should exceed 100 observations, while others suggest sample sizes larger than 200 or even 400 (Reinartz et al. 2009). In practical terms, it is difficult and costly to obtain such a large sample size. To avoid problems related to sample size and the sampling distribution of key statistics, as commented above, we have applied the Bootstrap technique as data resampling method. According to Fan (2003), a good fit can be achieved using this method in spite of a small sample size. Although it is difficult to know what the minimum sample size should be, and the number of Bootstrap replicas that should be utilized to approximate the true sampling distribution, the estimated parameters have been stable. To ensure the robustness of the results, we calculated a posteriori the statistical power of the model as a whole (objective 1) and for the verification of each proposed link among effective communication, supportive relationships, and satisfaction from the scope of the relational coordination framework (objective 2), in both cases using the obtained *R*^2^ coefficient (Cohen 1988). We accepted the results of the tests when 1 − *β* was greater than 0.8 (that is, it should have an 80% or greater chance of rejecting or accepting properly the hypothesis). In any case, the emphasis of the SEM analysis must be placed on its ability to test the theorizations raised, rather than on the fit of the model.

## Results

### Contribution of effective communication and supportive relationships to the satisfaction of fishermen with MPAs

**Fig. 3:**
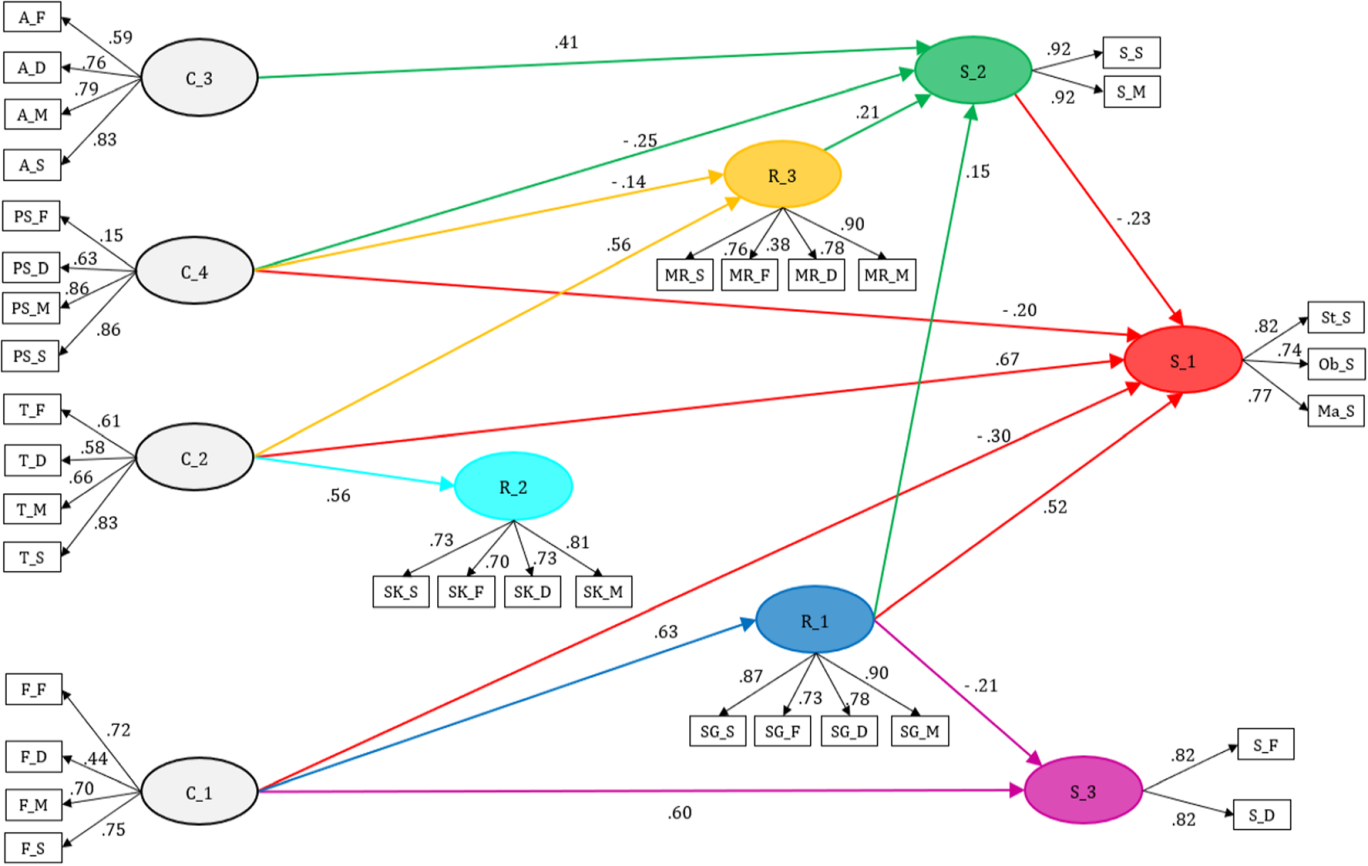
Best fitting structural equations model with the direct effects (standardized estimates) of effective communication and supportive relationships on fishermen satisfaction with MPAs

The best fitting SEM model for explaining the satisfaction of fishermen with MPA based on the relational coordination framework is presented in Fig. 3. Numbers on arrows represent standardized estimated coefficients for latent variables. The Satorra–Bentler scaled chi-square test was not significant, and the values obtained for RMSA (0.0643), NFI (0.950), and CFI (0.927) indicate that the model fit is acceptable. The statistical power (1 − *β*) for the fitness of the whole model equals 1.

In general, the latent factors were shaped by the observed variables of each dimension considered, except for the factors related to satisfaction, which separated the observed variables into three factors: satisfaction with the MPA (S1), satisfaction with managers (S2), and satisfaction with other users (S3). Thirty-five observed variables interacting with 10 latent variables were determined in the SEM model, including four exogenous variables and six endogenous variables. Effective communication factors (frequent communication (C1), timely communication (C2), accurate communication (C3), and communication for solving problems (C4)) were the exogenous latent variables, and supportive relationships factors (shared goals (R1), shared knowledge (R2), and mutual respect (R3)), and satisfaction factors (satisfaction with users (S3), satisfaction with managers (S2), and satisfaction with MPA (S1)) were the latent endogenous variables. Table 1 shows the goodness-of-fit statistics for the measurement model. Cronbach's alpha coefficients for latent factors ranged from 0.50 to 0.81, indicating that the internal consistency of the model is acceptable, following Ma et al. (2010). The average extracted variance (AVE) for latent factors ranged from 0.44 to 0.83, indicating that factors explain a relevant proportion of the variance and therefore that the convergent validity of the model is acceptable (Ahmad et al. 2016). The VIF oscillated between 1 and 4.12, indicating that there were no multicollinearity problems between the model’s factors.

**Table 1:** Goodness-of-fit statistics for the measurement model

Latent variable	Cronbach’s *α*	Average variance extracted
Frequent communication (C1)	0.53	0.441
Timely communication (C2)	0.57	0.564
Accurate communication (C3)	0.70	0.456
Problem-solving communication (C4)	0.54	0.468
Shared goals (R1)	0.67	0.530
Shared knowledge (R2)	0.81	0.679
Mutual respect (R3)	0.67	0.558
Satisfaction with MPA (S1)	0.67	0.607
Satisfaction with managers (S2)	0.50	0.835
Satisfaction with other stakeholders (S3)	0.80	0.667

The model explained 59% of the variability in the satisfaction with the MPA (S1) from five factors with a direct implication (C1, C2, C4, R1, S2) and six factors with an indirect implication (C1, C2, C3, C4, R1, R3). All the hypothesized factors, except shared knowledge (R2) and satisfaction with users (S3), intervened directly or indirectly in the model (Table 2). These results indicate that there is a strong relationship between the exogenous and endogenous variables. Contrary to our hypotheses, some factors had negative effects (C3, C4, R3, S2; Fig. 3). Effective communication and supportive relationships had a positive global impact on satisfaction with the MPA, while satisfaction with stakeholder groups had a negative global impact. The most important factors were timely communication (C2) and shared goals (R1) with a total positive effect of 0.642 and 0.489, respectively.

**Table 2:** Estimated parameters in the structural equation model

Structural equation	*R*^2^	*β*	Standard error	*P* value	VIF
R1	0.393				
C1		0.627	0.105	< 0.001	1.00
R2					
C2	0.316	0.562	0.110	< 0.001	1.00
R3	0.300				
C2		0.565	0.110	< 0.001	1.06
C4		− 0.144	0.122	0.006	1.06
S2	0.410				
C3		0.407	0.103	< 0.001	1.86
C4		− 0.249	0.075	0.001	1.10
R1		0.146	0.068	0.032	1.60
R3		0.210	0.104	0.043	1.39
S3	0.248				
C1		0.603	0.091	< 0.001	1.65
R1		− 0.212	0.100	0.034	1.65
S1	0.590				
C1		− 0.297	0.135	0.028	4.12
C2		0.668	0.106	< 0.001	4.02
C4		− 0.199	0.065	0.002	1.19
R1		0.522	0.071	< 0.001	1.73
S2		− 0.224	0.059	< 0.001	1.72

Three factors related with effective communication were significant for the supportive relationships factors. Shared goals (R1) was positively related to frequent communication (C1), shared knowledge (R2) was positively related to timely communication (C2), and mutual respect (R3) was positively related to timely communication (C2) and negatively to problem-solving oriented communication (C4). The model explained a range of 30–39% of the variability in the supportive relationships factors (statistical power 1 − *β*: 0.954). Therefore, L1 (*effective communication with the fishermen influence positively the quality of the fishermen's relationships with the groups interested in the MPA*) can be accepted.

About the repercussion of effective communication on satisfaction of fishermen with other MPA stakeholders (Table 2), the model explained 41.0% of the variability in satisfaction with managers (S2) and 24.8% in satisfaction with other users (S3) (statistical power 1 − *β*: 0.97). Frequent communication (C1) positively affected satisfaction with other users (S3), while satisfaction with managers (S2) was positively affected by frequent (C1), timely (C2), and accurate communication (C3), and negatively by problem-oriented communication (C4). Therefore, L2 (*Effective communication with the fishermen impacts positively on the satisfaction they perceive with the groups interested in the MPA*) can be accepted.

Three effective communication factors (C1, C2, and C4) contributed direct, positive, and significantly to satisfaction with the MPA (S1), explaining 59% of the variability (statistical power 1 − *β*: 0.999) when considering them altogether. Therefore, L3 (*Effective communication with the fishermen positively influences the perceived satisfaction with the MPA*) can be accepted. Considering them separately, frequent (C1) and timely communication (C2) positively affected satisfaction with the MPA (S1), while accurate (C3) and problem-solving oriented communication (C4) negatively affected it.

Mutual respect (R3) and shared goals (R1) positively affected satisfaction with managers (S2), while shared goals (R1) negatively affected satisfaction with other users (S3). Shared knowledge (R2) did not contribute significantly to satisfaction with other stakeholders. Therefore, L4 (*High-quality relationships with the fishermen positively influence the satisfaction they perceive from the groups interested in the MPA*) can be partially accepted (statistical power 1 − *β*: 0.988).

Supportive relationships factors altogether (R1, R2, and R3) had a positive contribution to satisfaction of fishermen with MPAs which allow us to accept L5 (*High-quality relationships with the fishermen positively influence their perceived satisfaction with the MPA*; statistical power 1 − *β*: 0.999). By other hand, considering them separately, shared goals (R1) and mutual respect (R3) contributed positively and negatively, respectively, to satisfaction with the MPA (S1), while shared knowledge (R2) did not intervene in the model.

Satisfaction with managers (S2) contributed negatively to satisfaction with the MPA (S1), while satisfaction with users (S3) did not intervene in the model. Therefore, L6 (*The satisfaction that fishermen feel with the groups interested in the MPA positively influences the satisfaction they feel about the MPA*) can be rejected (statistical power 1 − *β*: 0.865).

### Mechanisms of effective communication and supportive relationships that are beneficial for satisfaction with MPA

Overall, effective communication with fishermen benefits the quality of supportive relationships, the satisfaction with other stakeholder groups, and with the MPA (Table 3). In particular, the most beneficial dimensions of effective communication were frequent (C1) and timely (C2) communication, which positively affect four dimensions. Timely communication (C2) was the most beneficial factor for the model as a whole, with an important contribution to shared knowledge (R2), mutual respect (R3), and satisfaction with the MPA (S1), and a small positive effect on the satisfaction with managers (S2). Frequent communication (C1) was beneficial for shared objectives (R1) and satisfaction with other user groups (S3), and also slightly improved satisfaction with managers (S2) and with the MPA (S1). Accurate communication (C3) is the factor that most contributes to satisfaction with managers (S2), although it also has a slightly negative effect on satisfaction with the MPA (S1). Communication oriented to the solution of problems (C4), on the other hand, was the least beneficial factor for the model as a whole, slightly negatively affecting mutual respect (R3), satisfaction with managers (S2), and with the MPA (S1).

**Table 3:** Total standardized effects, direct (in italics) and indirect (in parentheses) identified in the structural equation model

	R1	R2	R3	S2	S3	S1
C1	0.627 (0.000) *0.627*	–	–	0.092 (0.092) *0.000*	0.470 (− 0.133) *0.603*	0.009 (0.306) − *0.297*
C2	–	0.562 (0.000) *0.562*	0.565 (0.000) *0.565*	0.119 (0.119) *0.000*	–	0.642 (− 0.027) *0.668*
C3	–	–	–	0.407 (0.000) *0.407*	–	− 0.091 (0.091 *0.000*
C4	–	–	− 0.144 (0.000) − *0.144*	-0.279 (-0.030) *-0.249*	–	-0.137 (0.062) − *0.199*
R1	–	–	–	0.146 (0.000) *0.146*	− 0.212 (0.000) − *0.212*	0.489 (− 0.033) *0.522*
R2	–	–	–	–	–	–
R3	–	–	–	0.210 (0.000) *0.210*	–	− 0.047 (− 0.047) *0.000*
S2	–	–	–	–	–	− 0.224 (0.000) − *0.224*
S3	–	–	–	–	–	–

The higher quality of relationships with the fishermen has also overall had a positive effect on satisfaction with objectives and with the MPA. The most beneficial relational dimension was shared goals (R1), which positively affected satisfaction with the MPA (S1) and with managers (S2), although it negatively affected satisfaction with other user groups (S3). Mutual respect (R3) improves satisfaction with managers (S2), although it slightly negatively affects satisfaction with the MPA (S1), while shared knowledge (R2) did not affect satisfaction at all.

## Discussion

Our results demonstrate that effective communication and supportive relationships are critical factors for explaining the satisfaction of fishermen with the MPA. The fitted model revealed that effective communication has a more significant influence than supportive relationships, although both components of cooperation have a positive influence. Fishermen's satisfaction with other stakeholder groups evidenced the occurrence of two latent factors: one that groups fishermen with managers and scientists, and another that groups them with divers and other fishermen. Our model better explains the satisfaction between fishermen and the managers than among fishermen and the other two user groups (other fishermen and SCUBA dive centers), suggesting that there are deeper underlying factors at play in the dynamic between these potential competitors (mostly SCUBA dive centers and scientists).

Specifically, frequent communication (C1) and the existence of shared objectives (R1) are the two critical mechanisms for generating satisfaction between fishermen and the other stakeholder groups but mainly with managers. Fishermen habitually maintain economic interests opposed to the other users of the MPA, particularly regarding other fishermen, with whom they generally compete for the same resources. They demonstrate negative attitudes towards one another, possibly because of the arrangement of MPA management in Spain. The country ostensibly strives to balance top-down authority with the grassroots organization of the cofradías, yet in practice the national government is dominant. At the same time, the regional governments assumed some management functions, and cofradías, in the end, only hold a consultative role (Hogg et al. 2017, 2021). A top-down management approach in an otherwise locally organized resource management scheme has possibly destabilized the authority of cofradías, diminishing their power to lead and organize fishing activities. The destabilization of the cofradías would in turn cause fishermen to lose incentives to follow the guidelines of the cofradías and maintain good relationships with other members of the cofradías. This possibility could explain the antipathy and lack of cooperation among fishermen.

This is a situation in which the existence of shared objectives negatively affects satisfaction of the fishermen with other users of the MPA. Here, sharing the same objectives poses a threat rather than an opportunity to take advantage of benefits derived from coordinated and cooperative management. The fact that fishermen depend on their incomes from harvesting, which tend to be reduced in the years immediately after the establishment of an MPA (Hilborn et al. 2004) results in their distrust of other users and their hesitance to collaborate, as has been reported in one of the MPAs included in this study (Hogg et al. 2017, 2021). As a result, fishermen do not consider it convenient or important to share knowledge with other users. The presence of competition also leads them to not recognize the MPA itself and the benefits that it could bring about (Pita et al. 2020), and entails one of the principal problems for those who deal with MPAs (Álvarez-Fernández et al. 2020).

In regard to the satisfaction of fishermen with managers, shared objectives are essential because fishermen want managers to prioritize fisheries conservation and work towards ensuring that the MPA generates higher catches for them with minimal restrictions. Spanish managers themselves favored this approach in framing MPAs as primarily of “fishing interest” (Revenga and Badalamenti 2008) to gain acceptance from fishermen during the MPA design and implementation process. We observed that fishermen feel that managers should provide them with the clear and accurate information needed to achieve higher catches without considering other factors such as ecological information (per. obs.). Hence, each component of effective communication has significant weight in the model explaining the satisfaction of fishermen with managers, above even supportive relationships.

On the other hand, when communication is oriented specifically to the resolution of problems, the satisfaction with managers diminishes. Several possibilities may explain this result. First, the resolution of problems may lead to actions that fishermen believe harm them finally, given that other objectives of an MPA (e.g., ecological) seem more important and are opposed to those of fishermen, and the resolution may inhibit fishing activities (such as new regulations that limit fishing activities). Another possibility is that this sort of communication primarily deals with conflicts involving fishermen, situations that—according to the opinion of the fishermen—are always resolved at the wrong time and to their detriment. Another fact that would explain this result is the assumption on the part of the managers that all relevant stakeholder groups must be treated the same way an assumption fishermen consider to be erroneous, given the differences in terms of their interests, dependency on resources, and their “natural rights” with respect to the environment and the resources that the MPA intend to regulate (Singleton 2009). During our surveys, we also recurrently observed that fishermen sought to gain an advantage over other stakeholders in the uses of the MPA, maintaining a constant victimizing posture (pers. obs.). Despite giving them leverage, they often persistently exert pressure on other users (mainly on divers) or continue to generate conflicts, which puts the viability and efficiency of the MPA at risk. This attitude results from the own social, economic, and territorial circumstance of each stakeholder group (McClanahan et al. 2005) and depend on the impact suffered by the various stakeholder groups as a result of an MPA’s establishment (McNeill et al. 2018).

The MPAs included in this study were proposed with an emphasis on fishery conservation objectives, using economic criteria related to the sustainable use of fisheries resources to evaluate MPA management and attending to the fishermen's interests to gain their acceptance. Although significant, the increase of biomass of target species and catches in the studied MPAs outside their boundaries is low (Tabarca and Cabo de Palos; Vandeperre et al. 2011) or undocumented (e.g., San Antonio, Serra Gelada, Cabo Tiñoso), with an explicit “edge effect” (Ohayon et al. 2021) that undermines the productive capacities of MPAs to regenerate the exploited fishery resources due to their small size. Furthermore, the lack of actual positive and sustained impact on the catches of the affected fishermen would explain the interest in establishing objectives to improve those catches and would be the cause of the fishermen's low satisfaction with the managers when resolving conflicts because these improvements in catches never materialized.

It is possible that cooperation will give rise to conflicts between different users (Gurney et al. 2016), a possibility that is evidenced in our study in the low variability explained by the satisfaction of fishermen with other stakeholders. In the case of the surveyed fishermen, we observed that they considered themselves the most-informed experts on the functioning of the marine ecosystem. Their aspiration has always been to be actively and directly engaged in the design of the MPA to apply their experience in that area, which they argue is the best knowledge to achieve efficient operation of the MPA. Some cognitive biases and self-interest are conditioning this position, and this is a reason which would justify their engagement in the management of the MPA in order to train them in the exercising of sustainable use of resources and developing more effective and appropriate strategies to achieve sustainable objectives that benefit them and society (Blythe et al. 2017) in an operating circumstances in which all stakeholder groups exchange meaningful knowledge. Some results evidenced that the inclusion of the local community nonetheless creates trust, which brings about improved conflict resolution, a sense of collective responsibility for marine conservation, increased socioeconomic benefits, and generally improved well-being of user groups and of the ecosystem as has been proposed since the beginning of the implementation of MPAs (Wells and White 1995), making fishermen part of the solution instead of a problem.

When fishermen share objectives with managers about the MPA their satisfaction with the MPA increases but not with other stakeholders. They do not see collective action as a relevant part of their profession, suggesting that what generates satisfaction with MPA is different from what generates satisfaction with other stakeholders. In our surveys we detected that they are not truly aware of the importance of sustainable use and conservation of the marine ecosystem nor of the collective governance of these common pool resources. They also carry out their activities in an individualistic way, as evidenced by our non-significant results related to the effect of shared goals and shared knowledge on satisfaction with other stakeholders. According to our results, timely communication and shared objectives are the principal aspects that affect the fishermen’s satisfaction with the MPAs, but from a very individualistic scope. Some studies (Di Franco et al. 2020) evidence that improved cooperation among fishermen, and between fishermen and managers, improves the management and governance of the MPAs and the socioeconomic condition of fishermen. Additionally, the identification and mitigation of all the negative impacts experienced by fishermen (e.g., uncertainty, stress) as opposed to solely those directly related to fishing restrictions or socioeconomic loss will also improve the effectiveness of MPA management (McNeill et al. 2018) and its legitimacy (Pollnac et al. 2010). Timely communication and shared objectives can aid to enforce both strategies by ameliorating the satisfaction of fishermen towards MPA.

There are examples that demonstrate that the combination of an adequate management plan and co-management involving directly local communities improve the cooperation among stakeholders and result in better outcomes from conservation efforts. These results may be explained both by the existence of policies and adequate means through the management plan (Voorberg and Van der Veeer 2020). For such co-management to be effective, our results evidence that there should be mechanisms of effective communication and adequate supportive relationships between fishermen and other user groups. In the case of the MPAs included in this study, stakeholder participation in MPA management is highly valued by the majority of users, effective communication being one of the principal modes of participation (Hogg et al. 2021). However, administrative bodies responsible for planning management of the MPAs included in this study tend to favor top-down approaches. Furthermore, according to the fishermen, the managers use their management proposals against them, ultimately resulting in further limitations on their activity, so they consider it more convenient to communicate as little as possible to resolve conflicts. This causes a backlash among stakeholders, particularly from affected fishermen (Yates 2014) which would also explain the negative effect of “communication oriented to the solution of problems (C4)” on fishermen satisfaction with managers.

A fact to reiterate is that the same fishermen that accepted the MPAs during their design and implementation, now, after some years of functioning, do not support their existence, despite that all the MPAs studied were implemented with objectives related to fisheries restoration. In the beginning, feelings of crisis produced by overfishing caused fishermen to view the MPA as a solution to their problems (Pollnac et al. 2001), circumstances in which the perception of collective risk raise the predisposition to accept the cooperation with other stakeholders and thresholds on the use of natural resources (Santos and Pacheco 2011). After a few years, however, the emergence of competition with other users exploiting the marine environment (e.g., diving centers; Hogg et al. 2018), the lack of an effective recovery of sustained captures with the creation of the MPA as discussed above, and disagreement with top-down regulations diminished their support of the MPA (Pita et al. 2020). This trend was exacerbated by the non-existence of effective communication and supportive relationships with fishermen, which drives the absence of agreement on common objectives and a deficient mutual respect, evidenced by our results. Resolving these issues related to key elements for an MPA's governance (Jones et al. 2014) are inexpensive to resolve and in other cases have quickly yielded positive results (Di Franco et al. 2020). Such a dynamic of ineffective communication and non-participation in MPA management promotes conflict and could bring about the destabilization of the MPA to the point of its collapse, preventing MPAs from providing the hoped-for ecosystem services for both the marine ecosystem and society (Jones and Long 2021). Earning the involvement of the fishermen and their support for the MPA management through timely communication with them, integrating their interests and objectives with those of the MPA, and accounting for their proposals would make them more compliant with MPA regulations and hence reduce conflicts (Chang et al. 2012).

## Conclusions

MPAs are considered key for the management of artisanal fisheries; and these fisheries, as part of a diversity of other activities and stakeholders, are considered key for the survival and resilience of many local communities. MPAs management is already complicated owing to the complex natural dynamics of the marine ecosystems they seek to conserve (Salas et al. 2019). The complexities derived from MPA management layered on top of this ecological complexity hamper the potency of MPAs as a conservation tool, both in an ecological and a socioeconomic context. In this sense, trying to reduce the malfunction of MPA management by reducing conflicts among users, and between users and the MPA, should be a main objective of management.

Applying a theoretical exploratory model to evaluate the links between effective communication, supportive relationships, and satisfaction of fishermen using MPAs, our results demonstrate that effective communication with fishermen significantly affects the supportive relationships of the fishermen with other users and the satisfaction of the fishermen with the MPA. Timely communication and shared objectives are the most important factors, with a direct and positive effect on the satisfaction of the fishermen with the MPA. However, mutual respect was negatively correlated with communication oriented to the resolution of problems. Our surveys evidenced that fishermen demand respect and accurate communication as part of MPA management, aspects that would be easy to implement in the management process without incurring additional economic costs. Managers and scientists should take into consideration that the more specific and accurate the terms used, the clearer and more comprehensible the message will be, the more accommodating communication channels will be, and communication will as a result be more effective in achieving satisfaction. By means of these mechanisms, the managers would achieve the effective integration of fishermen in MPA management and would improve management outcomes mainly by means of reducing conflicts.

Given the breadth of factors conditioning the MPA management and their success, these results are concerning. Among the many interconnected issues affecting the effectiveness of the MPA management, those related to user supportiveness are essential (Salas et al. 2019) and all of them are at least in part cultivated by effective communication and supportive relationships. Neglecting this components will drive to the MPA failure. Collaboration is an essential component for successfully implementing necessary management measures. The development of strategies based on shared objectives is a fundamental element for such collaboration, as is the development of supportive relationships through timely communication. All these key concepts (interconnected, collaboration, supportiveness, sharing) reflect the need to consider the interdependence between actors (and their actions) in the basis of the framework of MPA management. Individual behaviors influence the rest of the actors, or the knowledge or predisposition towards MPA of a single group of actors is insufficient to obtain maximum management efficiency. Both are reason enough to recognize this interdependence as an essential factor in the management of the MPA and propose the construction of coordinated productive networks—through sharing goals by timely communication in an authentic bottom-up co-management framework—to improve the desired performance outcomes for all the actors involved (Bolton et al. 2021).

These aspects should also be promoted and developed within fishing organizations to provide them with adequate leadership and shared messages through timely communication, in such a way that the positive effects of MPA management can be developed at the various scales on which the fishing sector is developed and depends. Integrating the functioning of every institution implicated in MPA management, and mainly those related to fishing, would help reduce the level of conflict between user groups and improve subsequently their governance and management (Cummings et al. 2020). Enforcing these mechanisms into the management will enhance the cooperation among stakeholders and ensure that MPAs increase their effectiveness.
